# Tectonic Graft for Persistent Leakage and Visual Outcome After Corneal Perforation Repair: A Case Series

**DOI:** 10.31729/jnma.5402

**Published:** 2020-09-30

**Authors:** Rachana Singh Rana, Leena Bajracharya, Reeta Gurung

**Affiliations:** 1 Tilganga Institute of Ophthalmology, Gaushala, Nepal

**Keywords:** *corneal perforation*, *laceration*, *siedel test*, *tectonic keratoplasty*

## Abstract

Keratoplasty is a modality of treatment for large and leaking corneal perforation in a tertiary center. We report cases of 20and 30-years old men presented in an emergency with history of road traffic accident 1 and 3 days back. Best corrected visual acuity was hand movement in both injured eye. Slit lamp examination of both cases revealed full thickness corneal laceration with Siedel test positive. Both cases underwent corneal laceration repair with resuturing and corneal glue on consecutive days but couldn't seal the leaking wound. Then ultimately both were undergone for tectonic keratoplasty. The final best corrected visual activity of involved eye was 6/36 in case 1 and 6/24 in case 2. Most cases of traumatic corneal perforation undergone urgent corneal repair but sometimes very difficult to seal the wound. In such cases donor cornea tissue may have to be used to maintain integrity of globe and better visual potential.

## INTRODUCTION

Corneal trauma can cause significant ocular morbidity and poor visual prognosis. Urgent diagnosis and management are required to prevent these complications. Corneal lacerations and perforations represent 6.8% to 14.7% of ocular traumatic injuries presenting in an emergency, although open globe injuries are uncommon.^1–3^

Eyes with corneal perforation need immediate treatment to preserve the anatomic integrity of the cornea and to prevent complications such as secondary glaucoma or endophthalmitis. Management of corneal perforation may range from temporary measures, such as application of bandage contact lens and glue application, to definitive treatment such as corneal transplantation.^4^

## CASE REPORT I

A 20 years old gentleman who was referred from outside Kathmandu valley with history of road traffic accident (RTA) 1-day duration. He sustained injury in the left eye (LE) with loss of vision and ocular pain. It was associated with redness and watering. No history of eye discharge. Patient had a complain of headache, no history of loss of consciousness or paranasal bleed. No history of diabetes mellitus, hypertension or any systemic illness. He was non vegetarian, doesn't consume alcohol. Best Corrected Visual Acuity (BCVA) in right eye (RE) 6/6 and left eye (LE) was HM. RE ocular examination revealed normal findings. LE ocular examination found normal eyelid and conjunctiva, but there was roughly about 3.5mm full thickness corneal laceration on nasal side involving 7-9 o'clock with prolapsed iris with Seidel I positive. There was flat anterior chamber and lenticular opacity. Posterior segment was difficult to be assessed. Eye ball was soft on digital tonometry. B-scan showed flat retina without any vitreous echo densities.

Systemic evaluation was unremarkable. X-ray orbit antero-posterior and lateral position showed no intraocular foreign body. Patient underwent emergency primary corneal repair. Cornea was sutured with 10-0 nylon. Oral antibiotics and painkiller were given on the day of surgery. In the first post-operative day of surgery, on examination Seidel I was positive from the repaired site and eye ball was in softer side. Immediately it was posted for resuturing. On the table resuturing and glue was tried but there was constant leakage. Tectonic penetrating keratoplasty was decided during surgery. Donor cornea was requested from eye bank which is within the premises of the hospital.

**Figure 1:**
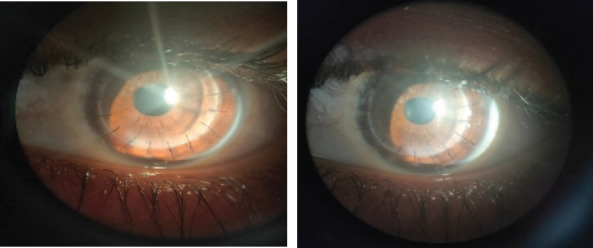
LE Post Keratoplasty with Grade 1 PSC.

Donor cornea size was 8 mm, size of Recipient cornea was 7.5 mm, slightly nasal eccentric graft with 16 interrupted 10-0 nylon was applied.AC was formed and there was no leakage at the end of procedure. Lenticular opacity was noticed. On the last follow up 10 months after keratoplasty, his left eye BCVA was 6/36. Graft was nasally eccentric but clear. There was posterior subcapsular cataract (PSC of grade 1) (Figure 1). Posterior segment was normal on dilated fundus examination.

## CASE REPORT II

Similar case of a 30 years old male presented with RTA of 3 days duration with injury in LE. BCVA in RE was 6/6 and LE was HM. LE had full thickness corneal laceration from 2-5 o'clock on temporal side with iris prolapse measuring 5 mm. AC was formed but siedel test was positive on applying gentle pressure on the globe (positive Siedel 2 test). There was mild AC reaction. Eyeball was in softer side. B scan of left eye was normal with no vitreous echodensities.

Patient underwent emergency primary corneal repair with 10-0 nylon but very difficult to seal the wound. The next day there was leakage from repaired site and eye ball was in softer side. Resuturing and glue was planned but it was not successful so emergency tectonic graft was carried out. Donor cornea size was 8.0mm and recipient cornea was 8.5 mm. Graft was taken eccentric temporally according to location of corneal laceration. Iris was excised from 4-5:30 o'clock.

**Figure 2:**
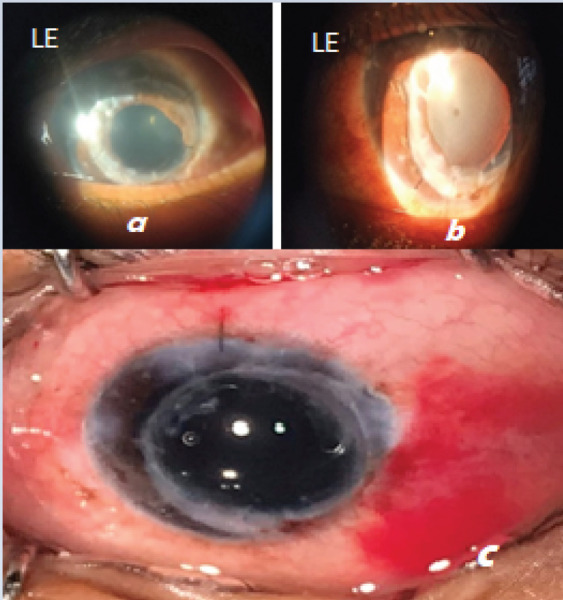
a) LE Postkeratoplasty (8 months) b) LE Postkeratoplasty with complicated cataract(16 months) BCVA 5/60 c) LE Post-Phacoemulsification and IOL implantation(BCVA 6/24 after 1 week)

Patient was followed up every month. On the last follow up BCVA in the operated eye was 6/36 with traumatic mydriasis. Patient presented with decreased vision for 2-3 months at 16 months of keratoplasty. VA LE 5/60, no improvement was there with refractive correction of +1.5 Dsph/-4.5.0 Dcyl at 10°. On examination he had developed complicated cataract. Then LE Phacoemulsificatio with posterior chamber (PC) IOL implantation was done (Figure 2).

## DISCUSSION

RTA are common occurrences every day. Patients younger than 33 years account for 82% of all the RTA ocular trauma. Ocular involvement in RTA may involve the eyelids, lacrimal canaliculi, orbital wall, conjunctiva, cornea, sclera, the extraocular muscles and corneal perforation accounts for 46.7% of all ocular injuries in this study.^5^

Depending on the size and location of the corneal perforation, treatment options include gluing, amniotic membrane transplantation, and corneal transplantation.

Nobe, et. al. have reported that for infectious corneal perforation if penetrating keratoplasty is performed grafts had a better chance to remain clear if surgery could be delayed for few days.^5^ However, if the surgeon feels that medical management or corneal gluing won't stop the aqueous leak from the site of perforation, a tectonic patch graft or large therapeutic graft should be performed at the earliest time possible. In cases with posttraumatic corneal perforation, primary closure should be carried out as soon as possible in order to prevent the ocular infection. In large posttraumatic perforations with irregular wound edge or tissue loss, primary closure may not seal the wound completely. While operating such cases, donor corneal tissue can be kept stand by to perform tectonic graft during the surgery. Our cases had shown good results with tectonic keratoplasty for traumatic corneal perforation. In both case report we found that final vision was 6/36 after keratoplasty.

In case, donor cornea is not immediately available, other options for treating such post-repair persistent leaks are corneal glue, conjunctival or amniotic membrane transplant and tenon's patch graft.^6^

One of the modalities of treatment of corneal perforation is tectonic keratoplasty if donor cornea is available, patient is young and posterior segment seems normal to maintain integrity of globe and better visual potential.

## Consent:

**JNMA Case Report Consent Form** was signed by the patient and the original article is attached with the patient's chart.

## Conflict of Interest

**None.**
